# Association of national and regional lockdowns with COVID-19 infection rates in Pune, India

**DOI:** 10.1038/s41598-022-14674-0

**Published:** 2022-06-21

**Authors:** Vidya Mave, Arsh Shaikh, Joy Merwin Monteiro, Prasad Bogam, Bhalchandra S. Pujari, Nikhil Gupte

**Affiliations:** 1 Pune Knowledge Cluster, Pune, India; 2 Johns Hopkins India, G-4 & G-5, PHOENIX Building, OPP. to Residency Club, Pune, 411001 India; 3 Indian Institute of Science Education and Research, Pune Campus, Baner Road, Pune, 411012 India; 4 Savitribai Phule Pune University, Pune, India

**Keywords:** Health care, Health policy, Health services, Public health

## Abstract

Assessing the impact of lockdowns on COVID-19 incidence may provide important lessons for management of pandemic in resource-limited settings. We examined growth of incident confirmed COVID-19 patients before, during and after lockdowns during the first wave in Pune city that reported the largest COVID-19 burden at the peak of the pandemic. Using anonymized individual-level data captured by Pune’s public health surveillance program between February 1st and September 15th 2020, we assessed weekly incident COVID-19 patients, infection rates, and epidemic curves by lockdown status (overall and by sex, age, and population density) and modelled the natural epidemic using the compartmental model. Effect of lockdown on incident patients was assessed using multilevel Poisson regression. We used geospatial mapping to characterize regional spread. Of 241,629 persons tested for SARS-CoV-2, 64,526 (26%) were positive, contributing to an overall rate of COVID-19 disease of 267·0 (95% CI 265·3–268·8) per 1000 persons. The median age of COVID-19 patients was 36 (interquartile range [IQR] 25–50) years, 36,180 (56%) were male, and 9414 (15%) were children < 18 years. Epidemic curves and geospatial mapping showed delayed peak of the patients by approximately 8 weeks during the lockdowns as compared to modelled natural epidemic. Compared to a subsequent unlocking period, incident COVID-19 patients were 43% lower (IRR 0·57, 95% CI 0·53–0·62) during India’s nationwide lockdown and were 22% lower (IRR 0·78, 95% CI 0.73–0.84) during Pune’s regional lockdown and was uniform across age groups and population densities. Both national and regional lockdowns slowed the COVID-19 infection rates in population dense, urban region in India, underscoring its impact on COVID-19 control efforts.

## Introduction

Worldwide, the trajectory of COVID-19 patients caused by SARS-CoV-2 has continued to rise since first detected in December 2019 in Wuhan, China^1–3^. As of May 1st, 2021, global patients exceeded 163 million with USA reporting the largest caseload and India—the world's second most populous country—recording the second largest cumulative caseload at 24.2 million^1^. With mounting morbidity and mortality in resource-rich and resource-limited settings alike^1,3–7^, scientists across the globe have developed vaccines and are investigating therapeutics and vaccines against COVID-19^8–12^. However, alternative non-pharmacological strategies remain critical to limit COVID-19 transmission.

Modelling exercises from India and elsewhere support the use of several such interventions to contain the spread of the COVID-19 pandemic, including complete lockdowns, curfews, regional containment strategies, social distancing, and the strict use of barrier protection and adhering to personal sanitary practices (i.e., gowning, use of masks, handwashing, etc.)^1–6,13–18^. Among these, lockdowns were most recommended by the World Health Organization^19^. Several European countries have adopted this strategy with relatively high success rates and more recent re-implementation. However, few assessments report the impact of lockdown in resource-limited settings, including India, which comprises large variations in urban and rural population density^20–24^.

Early in the COVID-19 pandemic, India instituted a nationwide lockdown for an extended 68-day period, which was followed by staggered phases of relaxation^7^. Notably, a rise in new confirmed patients prompted a second, regional lockdown in Pune city, a metropolitan city in western India. Using public COVID-19 surveillance data collected between February 1st and September 15th, 2020, we aimed to assess the real-world impact of lockdown on incident COVID-19 patients during the first wave in Pune city municipality and its subregions of variable population density. Further, we aimed to characterize the geospatial spread of the cumulative COVID-19 burden.

## Methods

### COVID-19 surveillance program in Pune, India

**Figure 1:**
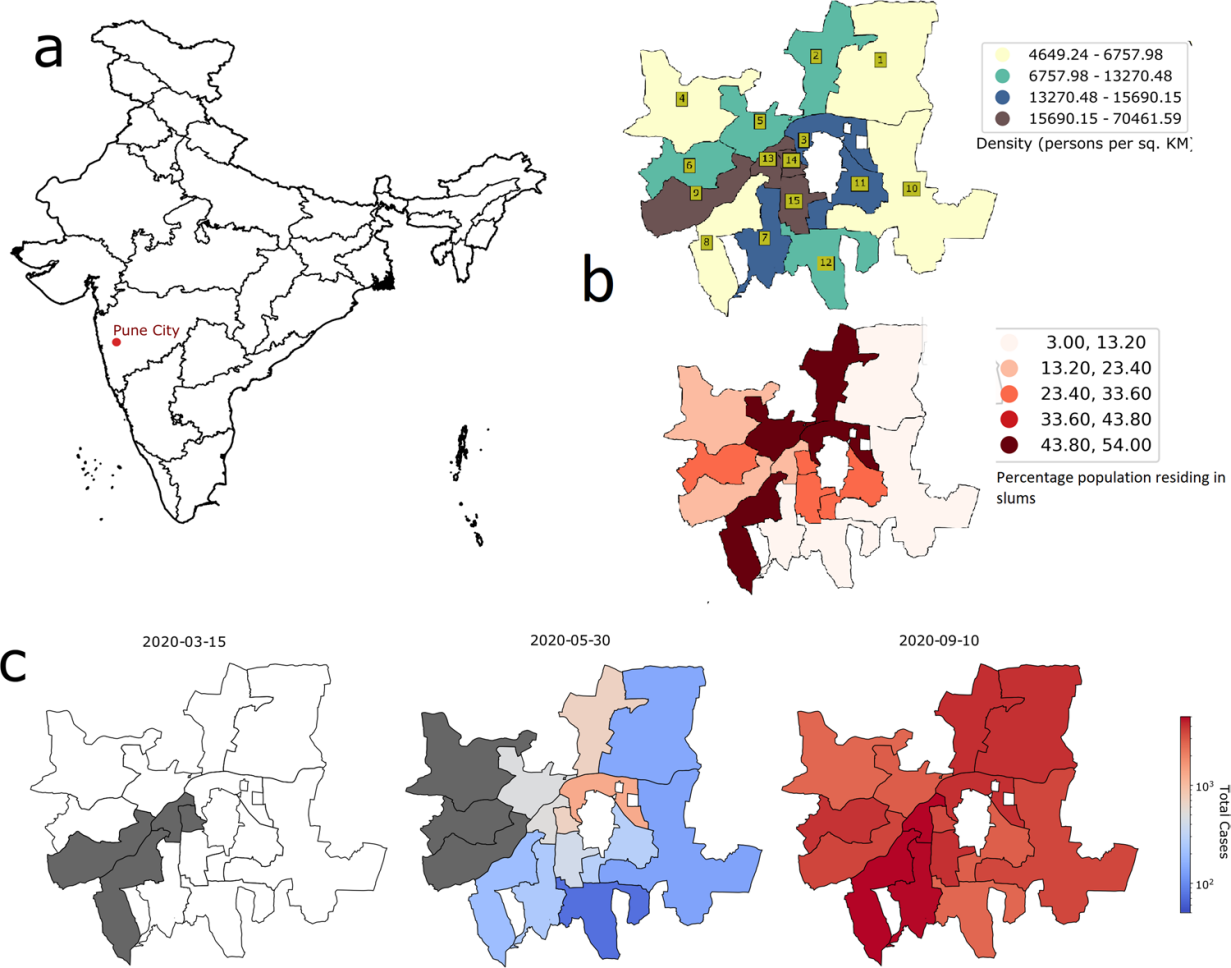
(**a**) Location of Pune City, India. (**b**) Geographic boundaries of ward offices located within Pune Municipal Corporation (PMC). Fill color indicates the quartile of population density (persons per square kilometer) and the proportion of slum population in each ward. Numbers inside the olive boxes indicate the official ward office number (see Supplemental Table S1 for the name corresponding to each ward office number). (**c**) the number of COVID-19 patients in each PMC ward office at beginning of the pandemic (left panel), at the end of the nationwide lockdown (middle panel), and at the end of the study period (right panel). The date is located at the top of each panel. Dark gray indicates < 50 patients, white indicates no patients, and the transition between blues and reds seen in the middle panel denotes approximately 600 patients.

Pune city-located in western India around 150 km east of Mumbai (Fig. 1a)—launched a COVID-19 surveillance program during the early stages of the pandemic (January 2020). Pune Municipal Corporation (PMC) collaborated with multiple public and private health facilities to establish SARS-CoV-2 diagnostics, quarantine facilities for asymptomatic persons, and hospital/critical care beds for moderate to severely ill patients diagnosed with COVID-19. In addition, community-based workers were mobilized to conduct contact tracing activities. A publicly accessible dashboard was established to report the cumulative COVID-19 caseload in the PMC’s 41 *Prabhags* (also known as electoral wards). The number tested and individual-level data, such as age, sex, residential address, COVID-19 test results, and COVID-19 outcomes, were centrally compiled on a regular (almost daily) basis^22^.

### Nationwide and Pune regional COVID-19 pandemic management

India’s initial response to the pandemic comprised travel advisories on international travel and suspension of visas from mid-January through mid-March. During this period, COVID-19 testing was administered to travelers who were returning from China and other foreign countries and had fever, cough or other viral respiratory symptoms^20^. Those testing positive were hospitalized for quarantine, and their close contacts were traced and underwent COVID-19 testing. The first nationwide lockdown was implemented from March 25th to April 14th, 2020 (Lockdown 1). Nearly all services and factories were suspended with reports of arrests for lockdown violations. During this time, Pune city expanded COVID-19 testing capacity, making testing available to persons with viral symptoms or within 14 days of COVID-19 exposure. The nationwide lockdown was extended from April 15th to May 3rd (Lockdown 2). Agricultural activities and essential services were allowed to function from April 20th, and Pune city areas were classified into red, orange, and green zones based on infection clusters. Red zones were defined by the central government based on case counts, doubling rate, and testing/surveillance findings. Initially, the central government defined the red zone as a particular area/district with more than 15 active cases. The area with < 15 cases with no recent surge were defined as the orange zone. The area with zero COVID cases were green zones. However later as the cases surged in the country, the central government allowed the states to categorize the zones. Notably, interstate transport was allowed for stranded individuals, and during the month of May alone, approximately one million migrants traveled via roads or trains to their home states, mostly from Maharashtra state. The lockdown was extended again from May 4th to May 17th (Lockdown 3), but with more relaxations in green zones where lower infection rates were reported. The final extension spanned May 18th to May 31st (Lockdown 4). States were given more authority to demarcate infection zones, and red zones were further divided into containment zones, which maintained stricter enforcement of lockdown norms than other zones.

The unlocking (resumption) of economic activities began in June 2020. During the first phase (Unlock 1, June 1st to June 30th), interstate travel was allowed with few state-specific restrictions while containment zones continued to follow lockdown norms. Phased unlocking continued in July (Unlock 2) when the authority to impose lockdowns was further decentralized to local governments. Pune city and the adjoining areas implemented a regional lockdown from July 14th to July 23rd in response to a sharp rise in COVID-19 patients. City and state authorities enforced a strict lockdown during the first week—a complete shutdown of all essential services, except emergency healthcare. This resulted in minimal movement in Pune’s public spaces. Slight relaxations in the supply of essential goods and services followed during the second week and Unlock 2 resumed in Pune on July 24th. August 1st to August 31st (Unlock 3) witnessed further relaxations in interstate travel and an end to nationwide curfews. Pune shopping malls and market complexes could remain open until evening, and cab services could operate with a restricted passenger load. However, lockdown restrictions continued in containment zones. During September (Unlock 4), gatherings of up to 50 persons were permitted while containment zones continued to follow lockdown norms. Early in September, Pune experienced a sharp rise in COVID-19 patients and became a top national COVID-19 hotspot (The lockdown events are summarized in the supplemental Fig. 1).

### Data curation

The area within PMC limits is divided into 15 administrative units, called ward offices (Fig. 1b), which are further divided into 41 electoral wards with similar populations, called *prabhags*. Individual-level data were included for the time period spanning February 1st to September 15th, 2020. According to daily press reports released by PMC, a total of 542,946 samples were collected for COVID-19 testing during the study period, and of these, 313,373 records were available. These data were curated to remove records with missing data. The remaining records were assigned to a *prabhag* using a machine learning based geocoder that was developed in house. The geocoding methodology is described in the supplementary material 1. Records with a confidence score below 0.5 out of 1.0 (provided by the ML geocoder) and records for persons residing outside PMC limits were removed. The final dataset used for this analysis comprises 241,629 records.

This analysis was done retrospectively on programmatic data without personal identifiers, hence individual patient consent was not obtained as infeasible. The Ethics Committee of Indian Institute of Science Education and Research, Pune, India approved the analysis of COVID-19 programmatic data and has waived the need for obtaining the consent. The analysis and reporting were performed in accordance with the relevant guidelines and regulations.

### Statistical analysis and mathematical modelling

The primary endpoint was weekly change in incident COVID-19 patients. The secondary endpoint was weekly infection rate; infection rate was calculated as the number of positive SARS-CoV-2 results divided by the total number of tests per 1000 population. Other endpoints included risk of COVID-19, defined as an incident COVID-19 case. Primary and secondary endpoints were assessed pre-lockdown, during lockdown and post-lockdown in the overall dataset and by population characteristics, namely sex, age group, and ward office-specific subcategories (population density and proportion residing in slum areas). Population density was calculated as number of people per 1 square kilometer and has been reported for all 15 PMC ward offices. For this analysis, population density was binarized as high (above the 3rd quartile of PMC ward office density, n = 6) or low-average (below the 3rd quartile of PMC ward office density, n = 9) (Fig. 1b). Since differences in infection rates existed among ward offices, the effect of lockdown on the primary endpoint was assessed using a multilevel Poisson regression model with random effects for ward office and test week. Change in the weekly infection rate over the study period was estimated using quasi-Poisson regression analysis. Logistic regression was used to assess the effect of risk factors on mortality. Epidemic curves for trends of incident patients over time were plotted using nonparametric locally weighted regression for the overall population and by sex, age group, and ward-specific subcategories.

We modelled the trajectory of the natural epidemic to estimate the delay of the peak of the pandemic. For this, we used a 9-compartmental model INDSCI-SIM that enables robust predictions taking into account the effects of various non-pharmaceutical measures (Supplementary appendix)^23,24^. There are a wide range of estimates for the value of *R*_*0*_; for example, Hilton and Keeling estimated *R*_*0*_ between 2 and 3^25^ while India specific study by Sinha found out the value to be around 1.8. In order to avoid overestimation of total patients, we also considered *R*_*0*_ =1.8^26^. Although there is no unique way to estimate actual number of patients, we assume infection on the first day (taken to be 1st April 2020) of the simulation to be three times reported patients. We note here that the choice of *R*_*0*_ and initial values may affect the final outcome, but our choices are conservative and more accurate estimation may make the results worse than reported here. We assessed the geospatial spread of COVID-19 patients over time and the visualizations were generated using the Python library geopandas (version 0.7.0, https://pypi.org/project/geopandas/0.7.0/). (Supplementary appendix). Data were analyzed in Stata Version 14·2.

## Results

### Population characteristics and risk of COVID-19

From February 1st to September 15th, 2020, of 241,629 SARS CoV-2 tests performed in all 15 PMC ward offices, 64,526 (26%) were positive, contributing to an overall rate of COVID-19 disease of 267·0 (95% CI 265·3–268·8) per 1000 persons. Among those diagnosed with COVID-19 disease, the median age was 36 (interquartile range [IQR] 25–50) years, 36,180 (56%) were male, and 9414 (15%) were children < 18 years. Compared to persons ages < 5 years, risk of contracting COVID-19 was higher among ages 5–18 years (incidence risk ratio [IRR] 1·11, 95% CI 1·06–1·17), 18–35 years (IRR 1·13, 95% CI 1·08–1·19), 35–50 years (IRR 1.2, 95% CI 1.23–1.35) and > 50 years (IRR 1·50, 95% CI 1.43–1.57) (Table 1).

**Table 1:** Estimated rate and incidence risk ratio of COVID-19 disease during the study period by population characteristics in Pune Municipal Corporation, India.

	Number Tested (N = 241,629)	COVID-19 Disease	
	n (%)	Rate per 1000 persons (95% CI)	Incidence risk ratio (95% CI)
Sex (N = 238,456)*			
Male	134,781 (57%)	268.4 (266.0–270.8)	Ref
Female	103,675 (43%)	267.4 (264.7–270.1)	1.01 (1.0–1.03)
Age (186,250)*			
< 5	6585 (4%)	283.6 (272.7–294.6)	Ref
5–18	23,754 (13%)	317.6 (311.7–323.6)	1.11 (1.06–1.17)
19–35	69,938 (38%)	315.0 (311.6–318.5)	1.13 (1.08–1.19)
36–50	49,152 (26%)	357.3 (353.0–316.5)	1.29 (1.23–1.35)
> 50	36,821 (20%)	415.1 (410.0–420.1)	1.50 (1.43–1.57)
**Population density, persons per 1 sq Km**			
High > 15,000	151,067 (63%)	263·4 (261.2–265.6)	Ref
Low-average < 15,000	90,562 (37%)	273·1 (270.2–276.0)	1.02 (0.89–1.18)
**Proportion residing in slum areas**			
> 50%	216,413 (90%)	264·6 (262.7–266.4)	Ref
< 50%	25,217 (10%)	288.4 (282.8–294.0)	1.12 (0.92–1.36)

*Available data.

### Lockdowns and incident COVID-19 patients

**Figure 2:**
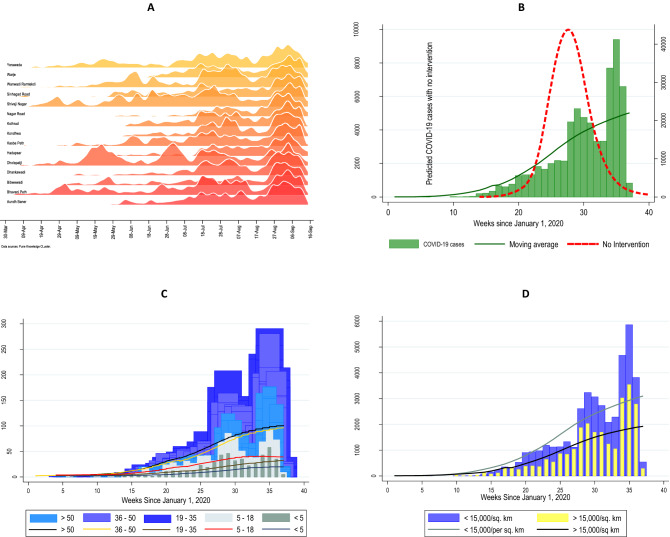
Shows the trajectory of new COVID-19 patients within Pune Municipal Corporation (PMC) over time. Lockdown/unlock periods defined as: pre-lockdown (1st February–24th March 2020); nationwide lockdown (25th March–31st May 2020); unlock 1–2 (1st June–13th July 2020); Pune regional lockdown (14th July–23rd July 2020); and unlock 2–4 (24th July–September 15th, 2020). (**A**) Number of daily incident COVID-19 patients across Pune Municipal Corporation ward offices during the study period. (**B**) Weekly incident COVID-19 patients by natural epidemic, and lockdown status in the overall patients. The red broken line represents projection modelling of the natural epidemic considering *R*_*0*_ =1.8; green line represents overall patients. (**C**) Weekly incidence of COVID-19 by age groups. (**D**) Weekly incident COVID-19 patients by population density.

Figure 2A,B illustrates the overall trajectory of new COVID-19 patients over the study period by lockdown status. In our model of the natural epidemic model, the epidemic would have reached the peak in mid- July as against to actual peak in mid-September in the absence of national lockdown. The approximate delay of the growth of the patients was 8 weeks (Fig. 2B). New COVID-19 patients maintained a steady rise before and during the nationwide lockdown with a 5% (1–8%) weekly increase in new infections during the lockdown (Table 2). This trajectory leveled off during the regional lockdown with a 7% (− 17 to 4%) weekly decrease in new infections (Table 2). Subsequent unlock periods (unlock 2–4) witnessed a sharp rise in incident COVID-19 patients with the largest weekly increase in new infections of 9% (7–10%) (Table 2). Incident patients peaked around the first week of September when Pune reported India’s largest burden of active COVID-19 disease (Fig. 2 A,B, *p* < 0.001) (Table 2).

**Table 2:** . Percent change (95% CI) in weekly incident COVID-19 infections by lockdown phase in the overall dataset and by population characteristics^a^.

	Pre-lockdown	Nationwide lockdown	Unlock 1–2	Pune lockdown	Unlock 2	Unlock 3–4
Overall	− 1% (− 18 to 19%)	5% (1–8%)	0.4% (0.2–3%)	− 7% (− 17 to 4%)	− 2% (− 11 to 8%)	9% (7–10%)
**Sex**						
Male	− 3% (− 17 to 14%)	3% (− 1 to 6%)	0.4% (− 2 to 3%)	− 6% (− 17 to 6%)	− 3% (− 17 to 15%)	10% (8–11%)
Female	− 4% (− 27 to 27%)	4% (− 0.3 to 7%)	0.3% (− 3 to 3%)	− 8% (− 17 to 3%)	1% (− 12 to 15%)	8% (6–10%)
**Age**						
< 18 years	38% (− 7 to > 95%)	2% (− 1 to 5%)	− 21% (− 23 to − 19%)	− 7% (− 17 to 4%)	− 8% (− 21 to 7%)	10% (8–12%)
≥ 18 years	− 2% (− 13 to 10%)	− 1% (− 2 to 1%)	2% (1–3%)	− 6% (− 10 to − 1%)	− 2% (− 8 to 5%)	9% (8–10%)
**Population density, persons per sq Km**						
High > 15,000	− 3% (− 18 to 16%)	− 2% (− 7 to 2%)	6% (2–9%)	− 14% (− 25 to − 1%)	− 8% (− 22 to 8%)	6% (3–9%)
Low < 15,000	− 3% (− 38 to 52%)	10% (5–14%)	− 2% (− 5 to 1%)	− 3% (− 22 to 20%)	2% (− 11 to 17%)	10% (8–12%)
**Proportion residing in slum areas**						
> 50%	− 14% (− 48 to 43%	12% (− 3 to 31%)	2% (− 6 to 2%)	− 8% (− 37 to 32%)	− 13% (− 37 to 20%)	9% (6–12%)
< 50%	1% (− 19 to 25%)	4% (1–7%)	1% (− 2 to 4%)	− 7% (− 18 to 5%)	1% (− 15 to 20%)	8% (7–10%)

^a^Pre-Lockdown (Feb 1–March 24, 2020); Nationwide Lockdown (March 25–May 31, 2020); Unlock 1–2 (June 1–July 13, 2020); Regional Lockdown (July 14–July 23, 2020); Unlock 2 (July 24–July 31, 2020); Unlock 3–4 (September 1–September 30, 2020).

Incident COVID-19 case trajectories by sex and age group illustrate a similar pattern (Fig. 2C, Supplementary Fig. 2) (Table 2). Ward offices with high population density (> 15,000 persons/square Km) had a significantly fewer new patients than wards with low-average population density (< 15,000 persons/square Km) (Fig. 2D , *p* < 0.001). During the nationwide lockdown, high population density areas had the lowest new infection rates with a 2% (− 7 to 2%) weekly decrease in new patients. Immediately following the Pune regional lockdown, areas with a majority living in slum areas had the lowest infection rate with a 13% (− 37 to 20%) weekly decrease in new infections (Table 2).

In multilevel Poisson regression models (Table 3), incident COVID-19 patients were 43% lower during the nationwide lockdown (IRR 0·57, 95% CI 0·53–0·62) and 22% lower during the Pune regional lockdown (IRR 0.78, 95% CI 0·73–0·84) compared to the post-regional lockdown period (unlock 3–4). Similar trends were observed across population characteristics, including sex, age, population density, and proportion residing in slum areas.

**Table 3:** Estimated effect of nationwide and Pune regional lockdowns on weekly incident COVID-19 patients using Poisson regression in Pune India, overall and by population characteristics^a^.

	Incidence Risk Ratio (95% CI)^b^	
	Nationwide Lockdown	Pune Regional Lockdown
Overall	0.57 (0.53 – 0.62)	0.78 (0.73 – 0.84)
**Sex**		
Male	0.58 (0.53 – 0.63)	0.74 (0.68 – 0.80)
Female	0.56 (0.51 – 0.62)	0.75 (0.69 – 0.82)
**Age**		
< 18 years	1.02 (0.99–1.05)	0.93 (0.83 – 1.04)
≥ 18 years	0.99 (0.98–1.01)	0.94 (0.90 – 0.99)
**Population density, persons per square Km**		
High > 15,000	0.54 (0.48–0.61)	0.70 (0.63 – 0.78)
Low average < 15,000	0.59 (0.53–0.65)	0.82 (0.75 – 0.90)
**Proportion residing in slum areas**		
> 50%	0.58 (0.46–0.74)	0.79 (0.63–0.98)
< 50%	0.57 (0.52–0.62)	0.78 (0.72–0.84)

^a^ Pre-Lockdown (Feb 1 to March 24, 2020); Nationwide Lockdown (March 25 to May 31, 2020); Unlock 1–2 (June 1 to July 13, 2020); Regional Lockdown (July 14 to July 23, 2020); Unlock 2 (July 24 to July 31, 2020); Unlock 3– (September 1 to September 30, 2020).

^b^ Compared to Unlock 3–4.

### Geospatial distribution of the cumulative COVID-19 burden

Among the 15 ward offices shown in Fig. 1b, the cumulative COVID-19 caseload steadily increased between pre-lockdown and post-lockdown phases (Supplementary Video 1). As illustrated in Fig. 1c, initial spread was primarily confined to a few ward offices with comparatively higher population density. By the end of the nationwide lockdown, western Pune continued to report a low case burden compared to central and eastern parts of the city. By the end of the study period, the caseload in all ward offices was above 2000, but the largest burden remained in the central part of the city aligned along the north–south axis.

## Discussion

During the early stages of the first wave of COVID-19 pandemic, India implemented a historic nationwide 68-day lockdown among 1.3 billion people and Pune region implemented a 10-day regional lockdown for its 3.1 million people following a brief period of unlock to curb a rapid increase in new COVID-19 patients^7,20^. By the week of September 1st 2020, India ranked second in the cumulative number of COVID-19 patients globally^1^, and Pune city had the country’s largest burden of active COVID-19 patients, overtaking other metropolitan cities^27^. This analysis of Pune city’s public health COVID-19 surveillance data found that the national lockdowns contributed to significant delay of the growth of patients by approximately 8 weeks. Furthermore, lockdowns flattened the COVID-19 pandemic curve with significant reductions in new patients and comparatively low infection rates during the nationwide and regional lockdown. Rapid expansion of patients was observed during later unlock periods corresponding to resumption of normal economic activities, following a pattern similar to countries that did not impose mobility restrictions^22^. Overall, this report provides valuable lessons to manage the crippling COVID-19 crisis being currently experienced worldwide.

The flattening of COVID-19 epidemic curves during nationwide and regional lockdowns was uniform across age groups and population densities. The greatest impact of the nationwide lockdown was observed among ward offices with high population density characterized by a 2% weekly decrease in new patients. The Pune regional lockdown appeared to be most effective among ward offices with > 50% of the population residing in slum areas, as evidenced by a 13% weekly decrease in new patients. Furthermore, early post lockdown period witnessed slower rise in weekly incident COVID-19 patients indicating the continued impact of lockdowns on maintaining social distancing. These findings are consistent with early modelling studies based on Indian nationwide data up to mid-April, which predict the effectiveness of strict social distancing for 42 days to reduce incident COVID-19 patients^13,18^. Population-wide data from Spain and Italy also demonstrate that strict lockdown was successful in flattening the COVID-19 epidemic curve^28^. However, this evidence may not be applicable to India and other resource-limited settings, as European lockdown periods were shorter at 14–21 days for earlier waves, and population densities and dynamics are significantly different^29^.

Consistent with earlier reports from India and other resource-rich settings, our analysis indicates that advancing age increases the risk of both contracting COVID-19 disease and mortality^2–7^. However, we did not find a sex-specific predilection for SARS COV-2 infection. We found that children < 5 years had the lowest rate of infection, yet our observed infection rate appears to be much higher than limited prior reports^2–7^. Notably, our geospatial visualization of the cumulative COVID-19 burden confirms the relatively rapid community-wide spread of the infection during the unlock periods. The average population density in Pune City, 5,600 persons per square kilometer, is more than tenfold higher than the average population density in India (464 persons per square kilometer)^29,30^. Thus, it is not surprising that the geospatial distribution of the COVID-19 burden was staggeringly rapid in Pune, particularly in the highest population density ward offices, which has contributed to India’s growing case counts.

Our report has a few limitations. We included only 44% of the original dataset after excluding records with missing data. Although nasopharyngeal swab PCR was the most common SARS CoV-2 diagnostic used in the public health system, around 35% of tests were done using rapid antigen testing. As the rapid antigen test is known to have high specificity and low sensitivity^31^, the case burden may have been underestimated when this testing method was used. Non-random ascertainment of COVID-19 patients is expected in programmatic setting, and this may adversely affect the impact of lockdowns. Also, migration of the population immediately after lockdown and its effect on the surge in COVID-19 cases could not be assessed due to the unavailability of data. Finally, contact tracing was conducted to rapidly identify and quarantine SARS-CoV-2 infected patients during the nationwide lockdown and this may have led to underestimation of the impact of lockdown on incidence of COVID-19.

In conclusion, as randomized controlled trial is not possible, this analysis of COVID-19 public health surveillance data assessing impact of lockdowns on spread of highly transmissible infection is very relevant for regions dealing with multiple devastating pandemic waves^1^. Importantly, while nationwide lockdowns may be counterproductive for economic reasons^32^, we provide important evidence for regional lockdowns as an effective public health measure to reduce the spread of SARS-CoV-2 infection. Our study adds confidence that non-pharmacologic interventions can be instated to limit transmission of infectious diseases in population dense, resource-limited settings. Further studies are needed to assess the epidemiology and effectiveness of lockdown in semi-rural and rural regions of India and beyond to inform successful, universal strategies for resource-limited settings worldwide.

## Supplementary Information

Supplementary Information 1.

Supplementary Video 1.

## Data Availability

The datasets generated during and/or analysed during the current study are available from the corresponding author on reasonable request. Please use the below link to request the data. https://docs.google.com/forms/d/18k1sFPuxS2I0K55tQBJFCRt-Twv0MqylWaestLPoRY8/viewform?ts=62037da8&edit_requested=true.
