# Supplementary material for: Association of national and regional lockdowns with COVID-19 infection rates in Pune, India
Source: Sci Rep. 2022 Jun 21;12:10446. doi: 10.1038/s41598-022-14674-0 (PMC9212203; doi:10.1038/s41598-022-14674-0)
Supplement: Supplementary file 1 — Supplementary Information 1. [file 41598_2022_14674_MOESM1_ESM.docx]

**Supplementary materials**

| **Sr No.** | **Supplementary material** | **Description** | **Page No.** |
| --- | --- | --- | --- |
| 1 | Geocoding methodology | Paragraph describing the geocoding methodology to process the addresses for geospatial mapping. | 2 |
| 2 | 9-compartmental model INDSCI-SIM | Details of model that provides robust predictions taking into account the effects of various non-pharmaceutical measures | 3 |
| 3 | Model to estimate Incident risk ratio | Explanation of the model used to estimate the incident risk ratio (IRR) and its equations. | 6 |
| 4 | Supplementary Table S1 | Official name and number corresponding to each ward office located within Pune | 7 |
| 5 | Supplementary Figure 1 | Incident COVID-19 case trajectory along with the COVID-19 lockdown events. | 8 |
| 6 | Supplementary Figure 2 | Weekly incident COVID-19 patients among males and females, respectively. Green line represents women and black line represents men. | 9 |
| 7 | Supplemental Material Video 1 | Video clip of geospatial mapping of daily COVID-19 patients over the analysis period. | 10 |

**Geocoding methodology**

Due to the unstructured nature of addresses in India, assigning an address to a geographical location is a challenging task.^22^ To understand the spatial evolution of the pandemic within PMC limits, we developed a machine learning model to process the address for each record and assign each address to a prabhag. To train the model, we used a database of 48000 addresses that had been manually assigned to their respective prabhags in PMC. We used 80% and 20% of the dataset for training and validation, respectively. To process the address, we created a database of localities and their respective prabhags. The addresses were simplified and normalised using a series of rules to minimise the noisy elements and were converted to a vector using a term frequency vectorizer. To emphasize certain unique localities within a prabhag, their frequency was multiplied by three. In the term frequency vectorization, we omitted tokens appearing rarely in the dataset. The threshold for this was chosen based on when the number of tokens on either side of the threshold are dramatically different. The threshold identified was 0·00003, yielding a vocabulary of ~5000 tokens. If the threshold was changed to ≥0·00004, the size of the vocabulary changed proportionally; if the threshold was decreased to 0·00002, the vocabulary more than doubled to ~12000 tokens, indicating the presence of many rare, “noisy” tokens. We used an ensemble of Multinomial Naive Bayes, XGBoost, and Random Forest classifiers with a Decision Tree classifier acting as a meta-classifier. With this architecture, we achieved an accuracy of 87%. Hyperparameters for Random Forest and XGBoost were chosen to reduce overfitting. The accuracy on the training set was ~91·5%, indicating slight overfitting. The reliability of the model was verified using 10-fold cross validation.

**9-compartmental model INDSCI-SIM**

**Compartmental model**

The work utilises INDSCI-SIM compartmental model [1] which is a nine compartmental model base on the work by Childs et al The compartments are susceptible $(S)$, exposed ($E$), asymptomatic infectious ($I^{a}$), pre-symptomatic infectious ($I^{p}$), mildly symptomatic infectious ($I^{m}$), severely symptomatic infectious ($I^{s}$), hospitalized ($H$), dead ($D$) and recovered ($R$). Transitions between these model compartments are shown schematically in Figure 1.


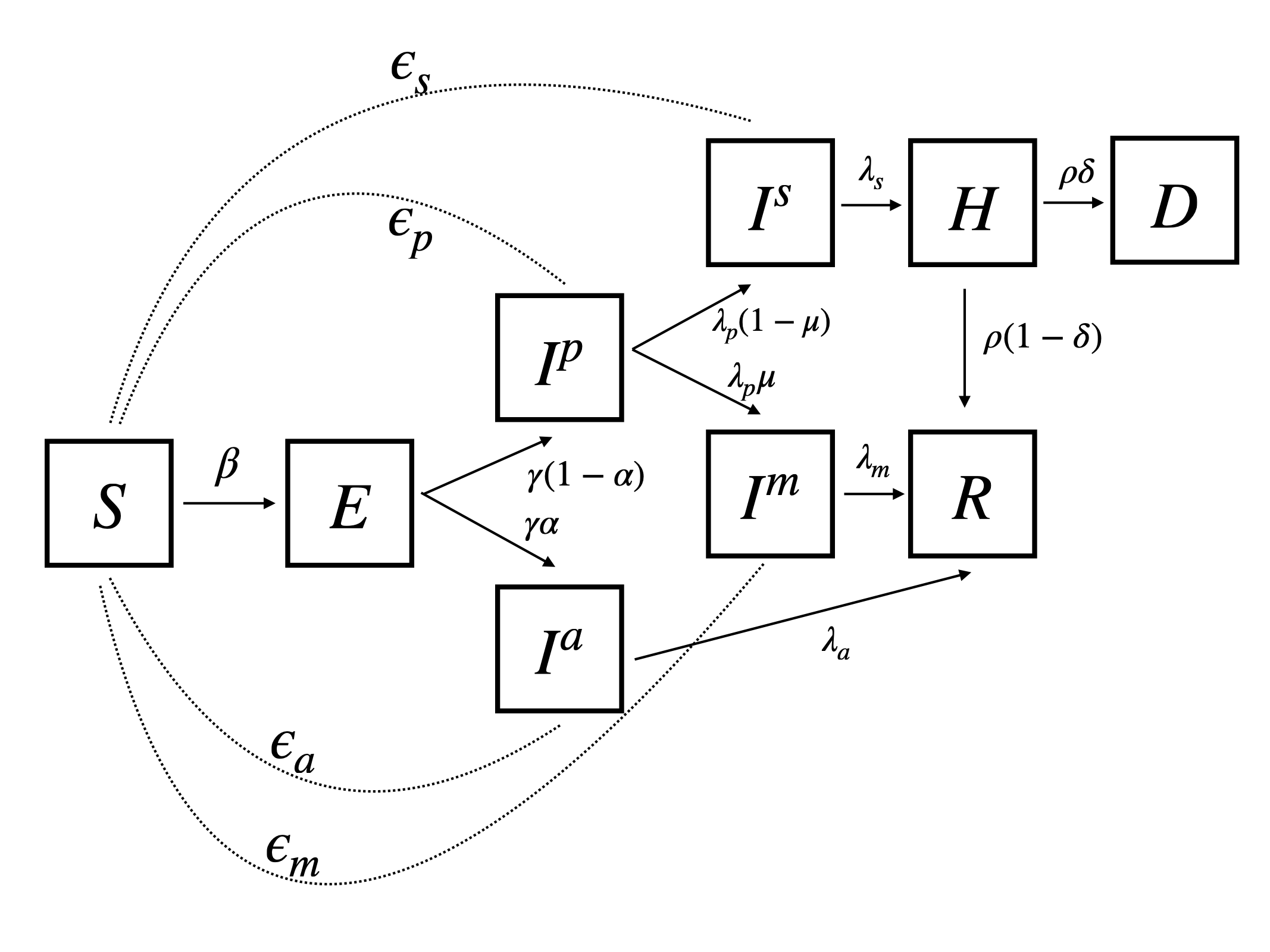


Figure 1: Schematic diagram of the compartmental model used in this analysis, adopted from [1,2]. The dotted lines indicate the force of infection from the infected compartments on the susceptible population. Transitions between compartments, denoted via solid lines and an arrow, are defined as in the text, which contains both bare rates as well as information as to how flow is to be divided between compartments. The number of persons transiting from one compartment to the other in each day depends on the product of the transition rates and the branching fractions.

The model equations, are:

$$\begin{matrix} \dot{S} & = & -\beta S(\epsilon_{a}I^{a}+\epsilon_{p}I^{p}+\epsilon_{m}I^{m}+\epsilon_{s}I^{s})/N, \\ \overset{̇}{E} & = & \beta S(\epsilon_{a}I^{a}+\epsilon_{p}I^{p}+\epsilon_{m}I^{m}+\epsilon_{s}I^{s})/N-\gamma E, \\ {\overset{̇}{I}}^{a} & = & \alpha\gamma E-\lambda_{a}I^{a}, \\ {\overset{̇}{I}}^{p} & = & (1-\alpha)\gamma E-\lambda_{p}I^{p}, \\ {\overset{̇}{I}}^{m} & = & \mu\lambda_{p}I^{p}-\lambda_{m}I^{m}, \\ {\overset{̇}{I}}^{s} & = & (1-\mu)\lambda_{p}I^{p}-\lambda_{s}I^{s}, \\ \overset{̇}{H} & = & \lambda_{s}I^{s}-\rho H, \\ \overset{̇}{R} & = & \lambda_{a}I^{a}+\lambda_{m}I^{m}+(1-\delta)\rho H, \\ \overset{̇}{D} & = & \delta\rho H \end{matrix}$$

The left-hand side of these equations denote first-order time derivatives. The population size is $N$. Infectious individuals in any associated compartment can infect the susceptible population regardless of symptoms and severity with a fixed transmission rate $\beta$. However, this quantity is modulated by the relative intensity of contacts between susceptible and infectious individuals whose effect is simply specified here through factors of $\epsilon$.

Of these parameters, the infectivity parameter $\beta$ is particularly central. It determines the effective reproduction ratio as the epidemic proceeds. The force of infection arising from asymptomatic cases alone is assumed to be lower in comparison to that arising from the pre-symptomatic, mildly symptomatic and severely symptomatic cases. The transmission coefficient can be computed using effective reproduction number $R_{0}$ (extracted from the field data) as

$$R_{0}=\beta\left( \frac{\alpha\epsilon_{a}}{\lambda_{a}}+(\alpha-1)\left( -\frac{\mu\epsilon_{m}}{\lambda_{m}}-\frac{\epsilon_{p}}{\lambda_{p}}+\frac{(\mu-1)\epsilon_{s}}{\lambda_{s}} \right) \right)$$

The values of parameters are adopted from and and are listed Table 1.

Table 1: The transition rates between compartments and the efficiency parameters. These parameters are fixed during the analysis.

| Parameter | Value (Rate (1/day)) | Description |
| --- | --- | --- |
| $\gamma$ | 0.5 | Transition rate from exposed to asymptomatic or pre-symptomatic (mean 2 days) |
| $\lambda_{a}$ | 0.1428 | Transition rate from asymptomatic to recovered (mean 7 days) |
| $\lambda_{m}$ | 0.1428 | Transition rate from mild to recovered (mean 7 days) |
| $\lambda_{p}$ | 0.5 | Transition rate from pre-symptomatic to |
|  |  | mild and severe (mean 2 days) |
| $\lambda_{s}$ | 0.1736 | Transition rate from severe to hospitalized (mean 6 days) |
| $\rho$ | 0.068 | Transition rate from hospitalized to recovered or dead (mean 15 days) |
| Parameter | Value (Fraction) | Description |
| $\alpha$ | 0.67 | Fraction of asymptomatic cases. |
| $\delta$ | 0.2 | Fraction of hospitalized that die |
| $\mu$ | 0.956 | Fraction of pre-symptomatic to mild cases. |
| Parameter | Value (Efficiency) |  |
| $\epsilon_{a}$ | 0.67 | Relative intensity of contacts for asymptomatic |
| $\epsilon_{p}$ | 1 | Relative intensity of contacts for pre-symptomatic |
| $\epsilon_{m}$ | 1 | Relative intensity of contacts for mild |
| $\epsilon_{s}$ | 1 | Relative intensity of contacts for severe |

**References**

[1] D.K Hazra, B. S. Pujari, S. M. Shekatkar, F. Mozaffer, S. Sinha, V. Guttal, P. Chaudhary, and G. I. Menon. The INDSCI-SIM model for COVID-19 in India. medrxiv, https://doi.org/10.1101/2021.06.02.21258203.

[2] Childs ML, Kain MP, Kirk D, Harris M, Couper L, Nova N, et al. The impact of long-term non-pharmaceutical interventions on COVID-19 epidemic dynamics and control. medRxiv. 2020;doi:10.1101/2020.05.03.20089078.

**Model to estimate Incident Risk Ratio (IRR)**

A multilevel Poisson Regression model was used to assess the changes in the newly diagnosed COVID-19 infections and differences by selected risk factors. The models would estimate IRRs, that can be interpreted as additional risk of COVID-19 infection in presence of a risk factor when compared to the referent group. We used the Poisson model at two levels. Level 1 was testing week and level 2 was the ward in which the tests were performed. Following is the model for a single risk factor.

PENALIZED QUASI LIKELIHOOD (PQL) PARAMETER ES

Level 1 Poisson Models

$$\log\left( \mu_{ij} \right)= \beta_{0j}+{\beta_{1}X}_{1j}$$

Level 2 Poisson Models

$$\beta_{0j}= \gamma_{00}+ \gamma_{0i}X_{1j}+u_{0j}$$

Poisson Mixed Models

$$\log\left( \mu_{ij} \right)= \beta_{0j}+{\beta_{1}X}_{1j}$$

$$= \gamma_{00}+ \gamma_{0i}X_{1j}+u_{0j}+ {\beta_{1}X}_{1j}$$

Where i = ward 1, 2, 3, …, nj and j = visit week 1, 2, 3, …, m

These univariable models were fitted for all the risk factors.

**Supplementary Table S1.** **Official name and number corresponding to each ward office located within Pune Municipal Corporation.**

| **Ward Number** | **Ward Name** |
| --- | --- |
| 1 | Nagar Road Vadgaonsheri |
| 2 | Yerwada-Kalas-Dhanori |
| 3 | Dhole Patil Road |
| 4 | Aundh-Baner |
| 5 | Shivajinagar-Ghole Road |
| 6 | Kothrud-Bawdhan |
| 7 | Dhankawadi-Sahakarnagar |
| 8 | Sinhagad Road |
| 9 | Warje-Karvenagar* |
| 10 | Hadapsar-Mundhawa |
| 11 | Wanawadi-Ramtekdi |
| 12 | Kondhwa-Yewalewadi |
| 13 | Kasba-Vishrambagwada* |
| 14 | Bhawani Peth* |
| 15 | Bibwewadi* |

*Denotes highest quartile of population density.

**Supplementary Figure 1:** Incident COVID-19 cases trajectory along with COVID-19 lockdown events


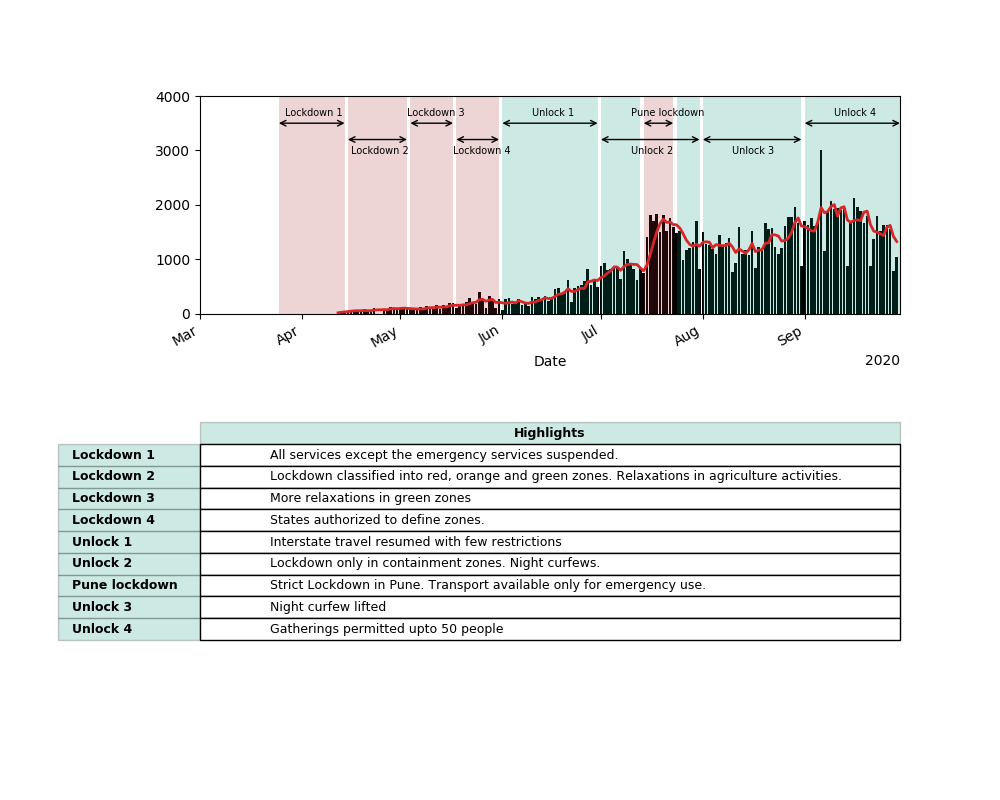


**Supplementary Figure 2:** Weekly incident COVID-19 patients among males and females, respectively. Green line represents women and black line represents men.

**Supplementary Material Video 1. Video clip of geospatial mapping of daily COVID-19 patients over the analysis period.**
